# Open Science Indicators as Metadata Fields?

**DOI:** 10.3389/frma.2021.768428

**Published:** 2021-11-11

**Authors:** Chris Fradkin, Rogério Mugnaini

**Affiliations:** ^1^ Psychological Sciences, University of California, Merced, Merced, CA, United States; ^2^ Institute of Psychology, Rio de Janeiro State University, Rio de Janeiro, Brazil; ^3^ School of Communication and Arts, University of São Paulo, São Paulo, Brazil

**Keywords:** open science, open science badges, metadata, transparency, scientific rigor

## Abstract

Since 2000, there has been qualitative growth in the field of scientometrics. Innovations such as the DOI and the ORCID have irrevocably changed the scientific landscape. They have enabled analyses previously unheard of, in the decades preceding the new millennium. This paper proposes open science indicators (open data, open material, preregistration) as article-specific metadata fields. The authors reference the history of funding information, from bare acknowledgements to metadata field. The authors describe the mission of the Center for Open Science, and its TOP Factor database, as well as the performance of open science badges. Possibilities for a pilot study are explored, with an acknowledgement of the complexity of this undertaking.

## The Scientometric Landscape

Since the electronic indexing of scientific publications, there has been qualitative growth in the scientometrics field. Innovations such as the DOI (2000) and the ORCID (2012) have transformed the landscape of our science. They have enabled bibliometric analyses that would have been unheard of years before. New bibliographic data sources such as Crossref (2000), Dimensions (2018), and Microsoft Academic (2016) are now challenging Web of Science and Scopus for their turf. The scientometric landscape is unfolding over time, driven by multiple stakeholders (publishers, funders, authors), from heterogeneous fields. This paper sets forth the possibility of open science indicators as metadata fields, functioning on an article-specific level.

### Open Science

In 2015, Brian Nosek and 269 colleagues published the paper “Estimating the reproducibility of psychological science” (Open Science Collaboration, 2015), in which the authors attempted to replicate the findings of 100 psychology studies published in 2008 in three prestigious journals (*Psychological Science*; *Journal of Personal and Social Psychology*; *Journal of Experimental Psychology: Learning, Memory and Cognition*). Surprisingly, the authors found that while 97% of the original set of studies showed statistically significant effect sizes, this was only reproduced in 36% of the replicated studies. This disclosure rocked the foundations of the scientific community, as it questioned the viability of a large percentage of its published findings. In response, the Open Science (OS) movement was born. The movement drew upon assumptions from the five OS schools of thought (infrastructure, measurement, public, democratic, pragmatic; Fecher and Friesike, 2014) and distilled them into specific goals and aims.

The aims of the OS movement are to upgrade the accessibility, transparency, and rigor of scientific publication (Nosek et al., 2015). The key points are reproducibility and replication. As a means of communicating and quantifying these goals, the Center for Open Science (COS) established Transparency and Open Promotion (TOP) guidelines (Nosek et al., 2015). These guidelines specify “eight modular standards, each with three levels of increasing stringency” (Nosek et al., 2015). These standards assess: 1) citation of data, code, and materials, 2) transparency of data, 3) transparency of code, 4) transparency of materials, 5) transparency of design and analysis, 6) pre-registration of studies, 7) pre-registration of analysis plans, and 8) replication.

### Stakeholders

The recent expansion of the scientometric landscape is the product of three groups of stakeholders: scholarly publishers, individual authors, and funding bodies. The convergence of their activities has altered the global research infrastructure. The interaction between these entities is codependent and collaborative, and serves the infrastructure as a whole. Innovations from past years set the stage for the OS movement. The DOI and ORCID are two such innovations that were legitimized in bibliographic metadata. Their legitimization was a collaboration between these groups of stakeholders. The same groups, and the same collaborative process, can legitimize OS indicators as bibliographic metadata fields.

### The Titans of Bibliographic Information^1^

Among citation databases, Web of Science (WoS) and Scopus are considered the most comprehensive and most trusted data sources. These “titans of bibliographic information” are regularly used for journal selection, research evaluation, and bibliometric analyses (Pranckutė, 2021, p. 1). Initially designed to facilitate global sharing of scientific knowledge, these databases now play key roles in academic hiring, resource allocation, education policy, and tenure (Aksnes et al., 2019; Kun, 2018; Rijcke et al., 2016). In WoS, journals are curated in the Core Collection, Current Contents Collection, and additional indices. Subscription cost is priced accordingly. In Scopus, similar content is available but with a single subscription fee and no room for modulation. Information from both databases is searchable through metadata fields, which include ORCID, DOI, and funding information. These fields facilitate search options; they impact different research cultures. Their adoption has been heterogeneous across disciplines and countries, as observed by Mugnaini et al. (2021) in relation to the DOI. Nonetheless, these recent innovations have reshaped the scientometric landscape.

### Funding Information and Its Impact on the Scientometric Landscape

An illustration of this reshaping can be seen in funding acknowledgements (FA), which are now accessible in WoS and Scopus metadata. These statements are typically one sentence in length, and provide acknowledgement of the research-funding source. In the 1990s, Cronin (1991) highlighted the significance of FAs in scholarly communication, and predicted its future use in scientometric studies. By later in the decade, Wellcome Trust’s Research Outputs Database (ROD) had organized funding sources from 214,000 biomedical articles (Dawson et al., 1998). Research on this trove provided evidence that articles including FA were likely to receive more citations than articles not reporting this information (Lewison and Dawson, 1998; Lewison et al., 2001; MacLean et al., 1998). In the 2000s, Giles and Councill (2004) developed an algorithm to extract and analyze FA information, and applied it to 335,000 documents in the CiteSeer computer science archive. Inclusion of FA was positively associated with citation count. In 2008, WoS began a systematic collection of FA data on funding text (FX), funding source (FO), and grant number (GN). In 2013, Scopus followed suit and began recording funding source (FUND-SPONSOR), funding source acronym (FUND-ACR), grant number (FUND-NO), and aggregated funding information (FUND-ALL) (Alvarez-Bornstein and Montesi, 2021). The inclusion of FA in these two mega-databases significantly expanded the vista of evaluative scientometric studies. In the 2010s, Díaz-Faes and Bordons (Diaz-Faes and Bordons., 2014) referred to FA indexation as a rich source of information and proposed systematic inclusion for the future. Since then, this new bibliographic field has gone through several further iterations, as is expected in such cases, under the scrutiny of the expert community (Alvarez-Bornstein and Montesi, 2021; Paul-Hus et al., 2016). Its evolution, however, is not limited to the expert community, as major funding bodies are increasingly mandating recognition of their contributions.

Another aspect of reshaping has been through public access mandates. These mandates were in response to the 2013 memorandum titled “Increasing Access to the Results of Federally Funded Scientific Research” (OSTP Memo; Holdren, 2013). Issued by the White House Office of Science and Technology Policy, the memo directed that all funding agencies with budgets over $100 million provide free access to their peer-reviewed publications. As of 2021, Google Scholar provides a public access section to their profiles, to help authors track and manage public access mandates for their articles (Sethi et al., 2021). These innovations are the product of the three main groups of stakeholders: publishers, individual authors, and funding bodies.

### Open Science Indicators as Metadata Fields

The inclusion of FA fields in databases enables funders to gauge the impact of their investment. This availability contributes to a more transparent culture: one held to higher standards. These standards are aligned with those of the OS movement: higher transparency, accountability, and scientific rigor (Nosek et al., 2015). In terms of values, FA information and OS practices could be sister indicators, although their movement occurs at different levels. FA is at the article level; OS practices are at the journal/publisher level. For OS practices to serve as OS indicators, they must be conceptualized at the article level. This contextual adjustment might be helped by following the template of FA field inclusion.

### Open Science Data

Since 2020, the COS has compiled data on the implementation of OS measures in their TOP Factor database (Center for Open Science, 2020). TOP Factor assesses journal policies for the degree to which they promote the eight OS norms of transparency and reproducibility. TOP Factor rates journal policies on a four-level scale, particular to each of the eight norms (Center for Open Science, n.d.-b). As of 2021, TOP Factor has tracked the implementation of OS measures among more than 900 signatories (Center for Open Science, 2021). In addition, TOP Factor tracks the implementation of OS badges. OS badges are visual icons displayed on the journal website; they spotlight transparency and scientific rigor.^2^ Badges signal to the reader that the content of an article (data, materials, pre-registration) is publically available and accessible in a persistent location (Center for Open Science, n.d.-a). As a promotional tool, OS badges have been found to be effective in incentivizing OS practices (Kidwell et al., 2016). Their implementation, however, has been lagging. As of 2021, of the more than 900 journals in the TOP Factor database, only 86 offered OS badges. Of these 86, only 19 journals displayed badges in a prominent position (i.e., in the table of contents).^3^ This figure could be higher, and we respectfully request that the COS consider adding a badge placement indicator to the TOP Factor scoring system.^4^

### The Operationalization of Open Science Indicators

The COS promotes OS norms at the journal/publisher level. This is evident in the makeup of TOP Factor, whose signatories are for the most part journals (Center for Open Science, 2021). By contrast, OS badges are article-specific; they have potential for scientometric usage. Think DOIs, FA information, lead author’s contact information. At an article level, metadata fields could contain information on the article’s open data, open materials, and pre-registration—the building blocks of OS.^5^ In its initial iteration, this information could be dichotomized (0 = *no*, 1 = *yes*, for open data, open material, preregistration). Further iterations could store repository information for open data, open material, and preregistration. For this task, the organization Crossref might be consulted. Crossref is a collective of academic publishers that is developing shared infrastructure to support more effective scholarly communications (Lammey, 2014, p. 84). One of their innovations, Funder Registry (FundRef until 2015), provides standardization for the reporting of funding sources for academic publications.

### Future Steps

OS badges are, in essence, OS indicators. They indicate an article’s compliance with OS standards. They perform this function at the article level, which makes them a valuable component for the execution of our plan. Our aim is to create dialogue about the possibility of OS indicators as metadata fields. To move forward toward our goal, articles must first be coded as to their meeting of OS badge requirements. As previously mentioned, they could be coded yes/no. As of 2021, there are less than 90 journals in TOP Factor issuing OS badges (Center for Open Science, 2021). This sample could be a starting point. With funding, we could devise a coding process, in collaboration with the editors and publishers of these journals. A pilot study of this sort could yield invaluable results, for the larger, long-term undertaking. Rough edges could be smoothed, realities fine-tuned. While these activities were in progress, the COS would be promoting OS standards among their signatories. With this parallel activity, the COS might notice that their influence was stronger, in recruiting journals to their cause. At that juncture, it would be helpful for the COS to implement a pipeline through which OS indicator information could flow. This would expand the breadth of the organization’s output, from handling journal-specific-only to article- and journal-specific information.

## Closing

OS research stands apart from other research in that it inadvertently promotes OS values. In that sense, every study examining OS standards keeps the buzzword of open science in the air. Every study published reminds us of the progress we have made, and of the many steps that lie ahead. The aforementioned pilot study could be a springboard of sorts; it could be a nexus for scholars who embrace OS values and wish to transform the research culture of the future. As of 2021, journal policies promote OS measures, although they do so to varying degrees (see Center for Open Science, n.d.-b). What is needed at this point is a core group of scholars, committed to the vision of legitimizing OS indicators as metadata fields.

We are aware of the challenges we face, in bringing this idea to fruition. We are aware of the time it took for FA information to be legitimized in bibliographic metadata—but in this digital age, we are hoping things run faster. We console ourselves that patience is required and that change does not happen overnight. Through this journey, our spirits are intact; we continue to follow our ideal. For a more transparent research culture, OS standards must move forward; OS indicators must move into the mainstream. They must be article-specific; they must be readily accessible; they must have metadata fields of their own.

## Footnotes

1 Attributed to R. Pranckutė’s (Pranckutė, 2021) “Web of Science (WoS) and Scopus: The Titans of Bibliographic Information in Today’s Academic World,” https://doi.org/10.3390/publications9010012.

2 OS badges are not machine-readable.

3 Badges displayed on the table of contents page receive significantly more views than those displayed in other areas.

4 A badge placement indicator would specifiy OS badge position: table of contents, individual article page, downloadable pdf.

5 Replication study status (yes/no) could also be contained in metadata fields.

## References

[B1] Aksnes D. W. Langfeldt L. Wouters P. (2019). Citations, Citation Indicators, and Research Quality: An Overview of Basic Concepts and Theories. Sage Open 9 (1), 2158244019829575. 10.1177/2158244019829575

[B2] Álvarez-Bornstein B. Montesi M. (2021). Funding Acknowledgements in Scientific Publications: A Literature Review. Res. Eval. 29, 469–488. rvaa038. 10.1093/reseval/rvaa038

[B3] Center for Open Science. (n.d.-a) Open Science Badges Enhance Openness, a Core Value of Scientific Practice. Available at: https://www.cos.io/initiatives/badges (Accessed August 27, 2021).

[B4] Center for Open Science. (n.d.-b) TOP-factor-rubric.docx (Version: 3). Available at: https://osf.io/t2yu5/ (Accessed August 29, 2021).

[B5] Center for Open Science (2020). New Measure Rates Quality of Research Journals’ Policies to Promote Transparency and Reproducibility. Available at: https://cos.io/about/news/new-measure-rates-quality-research-journals-policies-promote-transparency-and-reproducibility/ .

[B6] Center for Open Science (2021). top-factor.csv (Version: 22). [Data set]. Available at: https://osf.io/qatkz .

[B7] Cronin B. (1991). Let the Credits Roll: a Preliminary Examination of the Role Played by Mentors and Trusted Assessors in Disciplinary Formation. J. Documentation 47 (3), 227–239. 10.1108/eb026878

[B8] Dawson G. Lucocq B. Cottrell R. Lewison G. (1998). Mapping the Landscape: National Biomedical Research Outputs 1988-95. 9, London: Wellcome Trust.9.

[B9] Díaz-Faes A. A. Bordons M. (2014). Acknowledgments in Scientific Publications: Presence in Spanish Science and Text Patterns across Disciplines. J. Assn. Inf. Sci. Tec. 65 (9), 1834–1849. 10.1002/asi.23081

[B10] Fecher B. Friesike S. (2014). “Open Science: One Term, Five Schools of Thought,” in Opening Science. Editors Bartling S. Friesike S. (Berlin: Springer), 17–47. 10.1007/978-3-319-00026-8_2

[B11] Giles C. L. Councill I. G. (2004). Who Gets Acknowledged: Measuring Scientific Contributions through Automatic Acknowledgment Indexing. Proc. Natl. Acad. Sci. 101 (51), 17599–17604. 10.1073/pnas.0407743101 15601767 PMC539757

[B12] Holdren J. P. (2013). Memorandum for the Heads of Executive Departments and Agencies: Increasing Access to the Results of Federally Funded Scientific Research. Executive Office of the President, Office of Science and Technology Policy. Available at: https://obamawhitehouse.archives.gov/sites/default/files/microsites/ostp/ostp_public_access_memo_2013.pdf .

[B13] Kidwell M. C. Lazarević L. B. Baranski E. Hardwicke T. E. Piechowski S. Falkenberg L.-S. (2016). Badges to Acknowledge Open Practices: A Simple, Low-Cost, Effective Method for Increasing Transparency. Plos Biol. 14 (5), e1002456. 10.1371/journal.pbio.1002456 27171007 PMC4865119

[B14] Kun Á. (2018). Publish and Who Should Perish: You or Science? Publications 6 (2), 18. 10.3390/publications6020018

[B15] Lammey R. (2014). How to Apply CrossMark and FundRef via CrossRef Extensible Markup Language. Sci. Ed. 1 (2), 84–90. 10.6087/kcse.2014.1.84

[B16] Lewison G. Dawson G. (1998). The Effect of Funding on the Outputs of Biomedical Research. Scientometrics 41, 17–27. 10.1007/BF02457963

[B17] Lewison G. Grant J. Jansen P. (2001). International Gastroenterology Research: Subject Areas, Impact, and Funding. Gut 49 (2), 295–302. 10.1136/gut.49.2.295 11454809 PMC1728416

[B18] MacLean M. Davies C. Lewison G. Anderson J. (1998). Evaluating the Research Activity and Impact of Funding Agencies. Res. Eval. 7 (1), 7–16. 10.1093/rev/7.1.7

[B19] Mugnaini R. Fraumann G. Tuesta E. F. Packer A. L. (2021). Openness Trends in Brazilian Citation Data: Factors Related to the Use of DOIs. Scientometrics 126 (3), 2523–2556. 10.1007/s11192-020-03663-7

[B20] Nosek B. A. Alter G. Banks G. C. Borsboom D. Bowman S. D. Breckler S. J. (2015). Promoting an Open Research Culture. Science 348 (6242), 1422–1425. 10.1126/science.aab2374 26113702 PMC4550299

[B21] Open Science Collaboration (2015). Estimating the Reproducibility of Psychological Science. Science 349 (6251), aac4716. 10.1126/science.aac4716 26315443

[B22] Paul-Hus A. Desrochers N. Costas R. (2016). Characterization, Description, and Considerations for the Use of Funding Acknowledgement Data in Web of Science. Scientometrics 108 (1), 167–182. 10.1007/s11192-016-1953-y

[B23] Pranckutė R. (2021). Web of Science (WoS) and Scopus: The Titans of Bibliographic Information in Today's Academic World. Publications 9 (1), 12. 10.3390/publications9010012

[B24] Rijcke S. D. Wouters P. F. Rushforth A. D. Franssen T. P. Hammarfelt B. (2016). Evaluation Practices and Effects of Indicator Use-A Literature Review. Res. Eval. 25 (2), 161–169. 10.1093/reseval/rvv038

[B25] Sethi A. Hwang K. J. Verstak A. Acharya A. (2021). Track and Manage Your Public Access Mandates. Google Scholar Blog. Available at: https://scholar.googleblog.com/2021/03/track-and-manage-your-public-access.html .

